# Somatostatin receptor PET/CT in restaging of typical and atypical lung carcinoids

**DOI:** 10.1186/s13550-015-0130-2

**Published:** 2015-10-12

**Authors:** Vikas Prasad, Ingo G. Steffen, Marianne Pavel, Timm Denecke, Elisabeth Tischer, Konstantina Apostolopoulou, Andreas Pascher, Ruza Arsenic, Winfried Brenner

**Affiliations:** Department of Nuclear Medicine, Charité Universitätsmedizin, Berlin, Germany; Department of Hepatology and Gastroenterology, Charité Universitätsmedizin, Campus Virchow Klinikum, Berlin, Germany; Department of Radiology, Charité Universitätsmedizin, Berlin, Germany; Department of General, Visceral and Transplant Surgery, Charité Universitätsmedizin, Berlin, Germany; Institute of Pathology, Charité Universitätsmedizin, Berlin, Germany

**Keywords:** Lung carcinoids, Atypical carcinoid, Typical carcinoid, Somatostatin receptor, PET/CT, Management, Restaging

## Abstract

**Background:**

To assess the role of somatostatin receptor (SR) PET/CT using Ga-68 DOTATOC or DOTATATE in staging and restaging of typical (TC) and atypical (AC) lung carcinoids.

**Methods:**

Clinical and PET/CT data were retrospectively analyzed in 27 patients referred for staging (*N* = 5; TC, *N* = 4; AC, *N* = 1) or restaging (*N* = 22; TC, *N* = 8; AC, *N* = 14). Maximum standardized uptake value (SUVmax) of SR-positive lesions was normalized to the SUVmax of the liver to generate SUVratio; SR PET was compared to contrast-enhanced (ce) CT. The classification system proposed by Rindi et al. (Endocr Relat Cancer. 2014;21(1):1-16, 2014) was used for classification of patients in TC and AC groups.

**Results:**

Only 18/27 patients were found to have metastases on PET/CT. Of the 186 lesions, 101 (54.3 %) were depicted on both PET and CT, 53 (28.5 %) lesions only on CT, and 32 (17.2 %) only on PET. SUVratio of lesions was significantly higher in AC as compared to TC (*p* < 0.001). In patients referred for restaging, additional findings on PET lead to upstaging with change in management strategy in 5/22 (22.7 %) patients (AC, *N* = 5; TC, *N* = 1). In four patients (all AC) referred for restaging and in one patient (TC) referred for staging, additional findings on CT missed on PET lead to correct staging.

**Conclusions:**

Typical and atypical carcinoid patients have complex patterns of metastases which make it necessary to combine functional SR PET and contrast-enhanced CT for appropriate restaging. In patients referred for restaging SR, PET may have a relevant impact on treatment strategy in up to 22.7 of patients with typical and atypical lung carcinoids.

## Background

Neuroendocrine tumors (NET) of the lungs (LNET) represent approximately 30 % of all NET [1, 2] and account for 1–2 % of all lung tumors. According to current WHO classification, LNET are sub-classified into typical carcinoid (TC), atypical carcinoid (AC), and small cell and large cell neuroendocrine carcinoma (LCNEC). TCs are generally low-grade tumors, and ACs are intermediate-grade tumors, whereas the other two entities, small cell lung cancer (SCLC) and LCNEC, are high-grade neoplasms by definition with usually poor prognosis [3]. Of special note is the fact that up to 10 % of all lung tumors, especially, SCLC show neuroendocrine differentiation [4]. Diffuse idiopathic pulmonary neuroendocrine cell hyperplasia (DIPNECH) without any predisposing conditions has also been reported [5, 6]. DIPNECH is a disease with relatively indolent clinical course, usually remaining stable over several years but with the potential to metastasize in locoregional lymph nodes and rarely to extra thoracic sites [7].

The wide range of histopathological variations of NET with distinct prognosis often poses a clinical challenge not only with respect to the choice of therapy but also to the selection of the appropriate imaging tool for staging and restaging. For small cell lung cancer, the clinical role of F-18-fluoro-deoxyglucose (FDG) PET is well documented for patient management [8]. However, for the other histological subtypes of lung neuroendocrine neoplasms, there is no general consensus regarding the relative value of CT, MRI (of the liver and spine), and functional imaging with radiolabelled somatostatin analogs for staging and restaging. In specialized centers, patients with low- and intermediate-grade lung carcinoids like TC and AC [9] are usually imaged with somatostatin receptor (SR) scintigraphy or SR PET in addition to the conventional imaging procedures like CT and/or MRI. As yet, however, there has been only one prospective study examining the role of SR scintigraphy during the follow-up of patients after bronchial carcinoid resection [10].

Based on this background, we retrospectively analyzed all TC and AC patients referred to our ENETS Center of Excellence who had undergone both conventional contrast-enhanced CT imaging and SR PET/CT to evaluate if (a) SR PET and/or CT has an impact on the management of TC and AC, (b) to explore the correlation between SUVratio on tumor lesions and the histopathology, i.e., TC and AC, (c) compare SR PET and diagnostic CT in lesion detection, and (d) to look into the role of SR PET/CT in subset of DIPNECH patients.

## Methods

### Patient selection

Between 1.1.2008 and 13.2.2014, 36 patients with LNET were addressed for somatostatin receptor PET/CT; patients with aggressive LNET (SCLC, *N* = 1; LCNEC, *N* = 2) and those with unknown histopathology (n = 6) were excluded. The remaining 27 patients with histologically proven AC (*n* = 15) and TC (*n* = 12) were included in this retrospective analyses after approval by our local ethics committee (Charité Universitätsmedizin Berlin). All patients were followed up for a minimum of 6 months after the date of PET/CT.

PET/CT was performed in a total of 27 patients (18 females, 9 males) with TC + AC, for restaging after R0 (*N* = 20) and R1 resection (*N* = 2); in 5 patients, SR PET/CT was performed for primary staging purposes. Median age of patients was 63.6 years (range, 33.5–84.1 years). Three patients had secondary tumor manifestations (one patient with ileum NET, one patient with MEN1 syndrome, and one patient with prostate cancer). Patients’ characteristics are summarized in Table 1.

**Table 1:** Patients’ features: age is given as median/IQR and categorical variables are described by absolute and relative frequencies (%)

Parameter	Patients (*N* = 27)
Age (years)	63.6/53.0–71.0
Gender	
Female	18 (66.7 %)
Male	9 (33.3 %
Histopathology	
TC	12 (44.4 %)
AC	15 (55.6 %)
Initial TNM staging (available for 19 patients)	
T1	9 (47.4 %)
T2	9 (47.4 %)
T3	1 (5.3 %)
N0	15 (78.9 %)
N1	3 (15.8 %)
Nx	1 (5.3 %)
M0	12 (63.2 %)
M1	5 (26.3 %)
Mx	2 (10.5 %)
IASCL stage at initial diagnosis [27] (available for 19 patients)	
Stage Ia	8 (42.1 %)
Stage Ib	5 (26.3 %)
Stage IIa	1 (5.3 %)
Stage IV	5 (26.3 %)
Resection status	
R0	20 (74.1 %)
R1	2 (7.4 %)
Unresected	5 (18.5 %)

### Histopathology of lung carcinoids

Internal and external written histopathological reports were reviewed by an experienced pathologist (RA). In unclear or discordant cases, the tumor specimens were re-reviewed by our pathologist (RA) to establish a final diagnosis.

### Somatostatin receptor PET/CT

Ga-68 was eluted from Ge-68/Ga-68 generators and labeled either with DOTATATE or DOTATOC according to the respective standard labeling procedure already described elsewhere [11]. The selection of either DOTATATE or DOTATOC for imaging was purely based on the availability of the compound due to patent regulations. Ga-68-DOTATATE/DOTATOC PET/CT was performed according to the EANM Guidelines [12]. Mean radioactivity injected was 1.7 MBq/Kg of body weight, and the acquisition was performed 45–60 min after the injection of the radiotracer. Until June 2010, PET/CT was performed by a Biograph 16 PET/CT system (Siemens AG, Germany), five to six bed positions each with 3-min acquisition time. After June 2010, all PET scans were acquired in a 3-dimensional acquisition mode on a Gemini TF 16 PET/CT system (Philips Medical Systems) [13]. The standard 3D-LOR algorithm of the system software was used with default parameter settings to reconstruct transaxial slices of 144 × 144 voxels with 4.0 × 4.0 × 4.0 mm^3^; 10–12 bed positions each with 1.5-min acquisition time; CT was used for the attenuation correction for both the scanners. If contrast-enhanced multi-phase CT was performed at the time of PET/CT (*N* = 25), 70–100 ml Ultravist 370 (Bayer Schering Pharma, Berlin, Germany) with a delay of 30 s for the arterial phase, 50 s for the portovenous phase, and 70 s for venous phase was injected intravenously and images were acquired using bolus tracking methodology, with a collimation of 0.75 mm and a slice thickness of 16 × 0.75 mm for arterial and portovenous phase whereas for venous phase, slice thickness was 16 × 1.5 mm. In two patients, contrast-enhanced CT was performed within 4 weeks of PET/CT.

The somatostatin receptor expression in the tumor and normal liver tissue was semi-quantitatively assessed by calculating the maximum standardized uptake value (SUVmax). SUVmax for both the tumor region and the normal liver was determined by using a manual region of interest (ROI) in transaxial attenuation-corrected PET slices. The uptake in the liver was taken as reference value, and the SUVmax of the tumor lesions were normalized internally using SUVmax of the liver for normalization according to the formula normalized uptake in tumor (SUVratio) = SUVmax tumor / SUVmax liver.

SUV were measured only for those lesions which were definitely positive by visual assessment, i.e. the uptake of the lesion was higher than the uptake of the immediate normal surrounding tissue, and which had a size of more than 10 mm in diameter. For bone lesions, size was not taken into consideration according to RECIST criteria. The SUVmax values of Ga-68-DOTATATE/DOTATOC PET/CT can theoretically be influenced by several factors like difference in scanner type, acquisition and reconstruction parameters, and differences in the peptide affinity towards somatostatin receptors among others. For these reasons the normalized values (SUVratio) were preferred over SUVmax for describing the characteristics in the degree of somatostatin receptor expression in both metastases and the primary tumors.

### Image analyses

The PET/CT images were analyzed in an interdisciplinary tumor board by experienced and board-certified physicians, primarily by a radiologist (TD), and a nuclear medicine physician (VP). For the image re-evaluation of this study, consensus of the two main readers, nuclear medicine physician (VP), and radiologist (TD) was considered sufficient. In case of discrepancy between these two readers, a second nuclear medicine physician (WB) was involved for a final decision. Data were put in clinical perspective with the pathologist (RA), the attending gastroenterologist (MP), and the surgeon (AP). Lesions seen on PET/CT were characterized as tumor tissue or metastases only if all the physicians achieved a common consensus; in case of any discrepancy between the panelists, lesions were followed up with CT and/or MRI and by the clinical course. A tracer accumulation on PET images was defined as positive tracer uptake by visual assessment by the two observers VP and TD. Lesions detected only by one modality (CT or PET) were termed positive or negative based on follow-up or complementary imaging modalities like MRI and/or CT. Those patients having both receptor-positive lesions as well as receptor-negative lesions appreciable on CT only were classified as having “mixed lesions”.

### Statistical analyses

The R-software (version 3.1.3, R Foundation for Statistical Computing, Vienna, Austria) was used for statistical calculations. Categorical variables were analyzed using contingency tables and chi-squared test. If the absolute frequency in contingency table cells was ≤5, Fisher’s exact test was used. According to histograms and quantile-quantile plots, a non-parametric distribution of metric variables (SUVmax, SUVratio) was assumed and descriptive parameters are given as median, interquartile range (IQR; 25th quantile-75th quantile), and range (minimum-maximum). Differences between unpaired groups were analyzed using the non-parametric Kruskal-Wallis test (>2 groups) and the Mann-Whitney *U* test (2 groups), respectively. The association of a metric and a dichotomous variable was analyzed using receiver-operating characteristics (ROC) curves. The optimal cutoff value was defined by the point on the ROC curve with the minimal distance to the point with 100 % sensitivity and 100 % specificity. All tests were performed as two-sided tests, and *p* values of less than 0.05 were considered as significant.

## Results

### Histopathology

Patient’s histopathology was classified according to the grading system proposed by Rindi et al. [14]. The major difference between the classification proposed by Rindi et al. and the WHO classification is the cutoff value of Ki67.

Based on the Rindi et al. classification, the patient series comprised 12 TC (44.4 %) and 15 AC (55.6 %) patients.

**Fig. 1:**
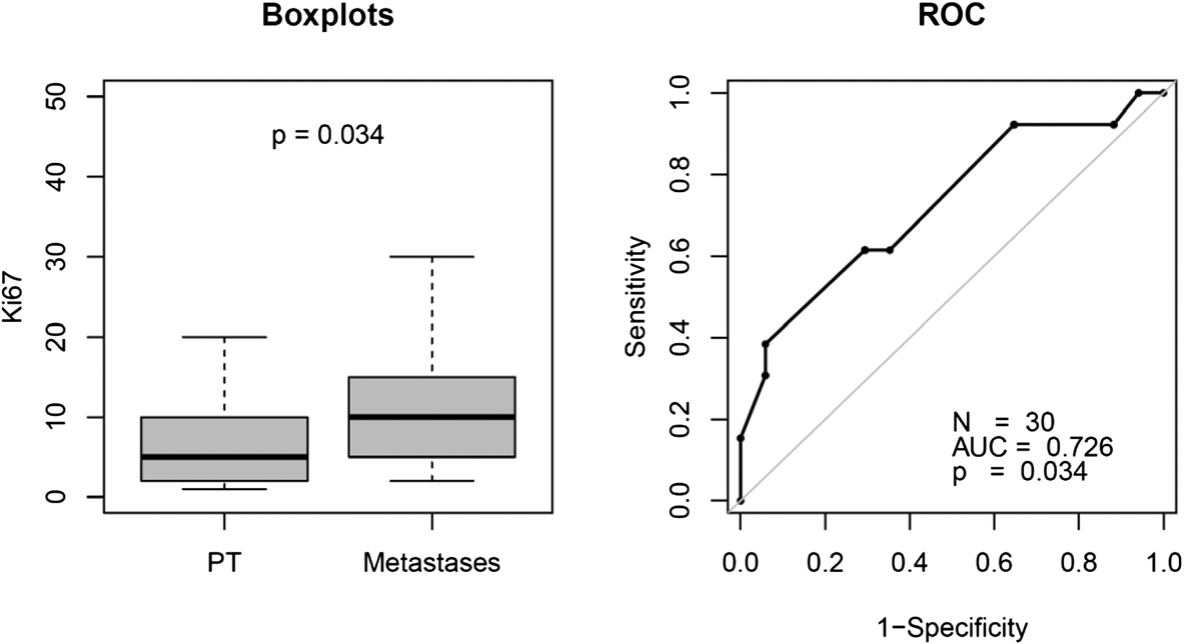
Ki67 of primary tumor (PT) and metastases depicted as boxplots and receiver operating curves (ROC). Proliferation rates in PT (*N* = 13) were significantly lower compared to metastases (*N* = 17)

Assessment of Ki67 in tumor tissue (13 PT, 17 metastases) was available in 23 patients (8 TC, 15 AC). In six patients, Ki67 was available from different sites at different time points. The median proliferation rate (Ki67) in metastases (10.0; IQR, 5.0–15.0; *N* = 17) was significantly higher compared to primary tumors (5.0; IQR, 2.0–10.0; *N* = 13) (*p* = 0.035) (see Fig. 1). The median time interval of 31.9 months (IQR, 17.2–44.1) between SR PET and Ki67 evaluation in specimens was relatively long, which could have been partially responsible for the aforementioned significant difference in the Ki67 of metastases and primary tumor.

### Imaging

#### PET vs. CT—lesion-based analyses

Because of the retrospective nature of the study and ethical issues, none of the discordant lesions were histopathologically confirmed. The discrepant lesions between PET and CT were confirmed by clinical follow-up for at least 6 months and wherever needed also with correlative imaging (CT, MRI, or PET).

Overall, 186 lesions were analyzed: 29 lesions in lungs suspected to be primary tumors (*N* = 6 patients, 3 with multiple lung nodules subclassified as DIPNECH), bone 52, LN 29, liver 49, and other metastases 27. One hundred one lesions (54.3 %) were concordant (both PET and CT visualized the lesions) whereas 53 (28.5 %) lesions were only visible on CT and 32 (17.2 %) lesions were only positive in PET (Table 2). Lesions only positive in PET were significantly more frequent in AC patients (30/148 = 20.3 %) compared to TC patients (2/38 = 5.3 %, *p* = 0.028).

**Table 2:** Absolute and relative frequency of concordant and discordant lesions on PET/CT

	Only positive on PET	Only positive on CT	Concordant positive on PET and CT	Total
PT^a^	0 (0 %)	21(72.4 %)	8 (27.6 %)	29 (15.6 %)
Liver	9 (18.4 %)	23 (46.9 %)	17 (34.7 %)	49 (26.3 %)
Bone	17 (32.7 %)	0 (0 %)	35 (67.3 %)	52 (28.0 %)
Lymph Node	2 (6.9 %)	9 (31.0 %)	18 (62.1 %)	29 (15.6 %)
Others	4 (14.8 %)	0 (0 %)	23 (85.2 %)	27 (14.5 %)
Total	32 (17.2 %)	53 (28.5 %)	101 (54.3 %)	186 (100 %)

^a^3 DIPNECH patients with multiple lung nodules also included

PET failed to detect 21/29 lung lesions. PET detected 9/49 (18.4 %) additional liver metastases (Table 3), which were not visible on CT. In contrast, CT picked up 23/49 additional liver lesions (46.9 %) not seen on PET (somatostatin receptor negative). One lesion seen on CT was later on classified as a liver cyst on biopsy. In this patient, all the lesions seen on CT had the same characteristics as the lesion biopsied and therefore were considered as cysts. Two additional lymph nodes (6.9 %) were seen on PET while CT picked up 9/29 (31 %) pathologically enlarged lymph nodes confirmed as metastases by follow-up. CT missed 17/52 bone lesions (32.7 %) whereas PET depicted all 52 bone lesions (results are summarized in Table 2).

**Table 3:** Patients’ characteristics with confirmed liver metastases on CT or PET in follow-up

	Patient 4	Patient 12	Patient 20	Patient 27	Patient 29	Patient 30	Patient 31	Patient 35
Ki67	15 %	10 %	5 %	10 %	1	10 %	20 %	7 %
Histo	AC	AC	AC	AC	TC	AC	AC	AC
Lesion size (mm)	7–32	14–40	20–150	–	–	15–62	21–23	15–19
Somatostatin receptor-positive lung lesions	19/24	0/5	5/5	1/1	2/2	0/7	2/2	2/2
CT-positive lesions	21/24	5/5	4/5	0/1	0/2	7/7	2/2	2/2
SUVmax	7.4–17.4		–	5.2–10.5			16.2–44.4	16.5–17.5

**Fig. 2:**
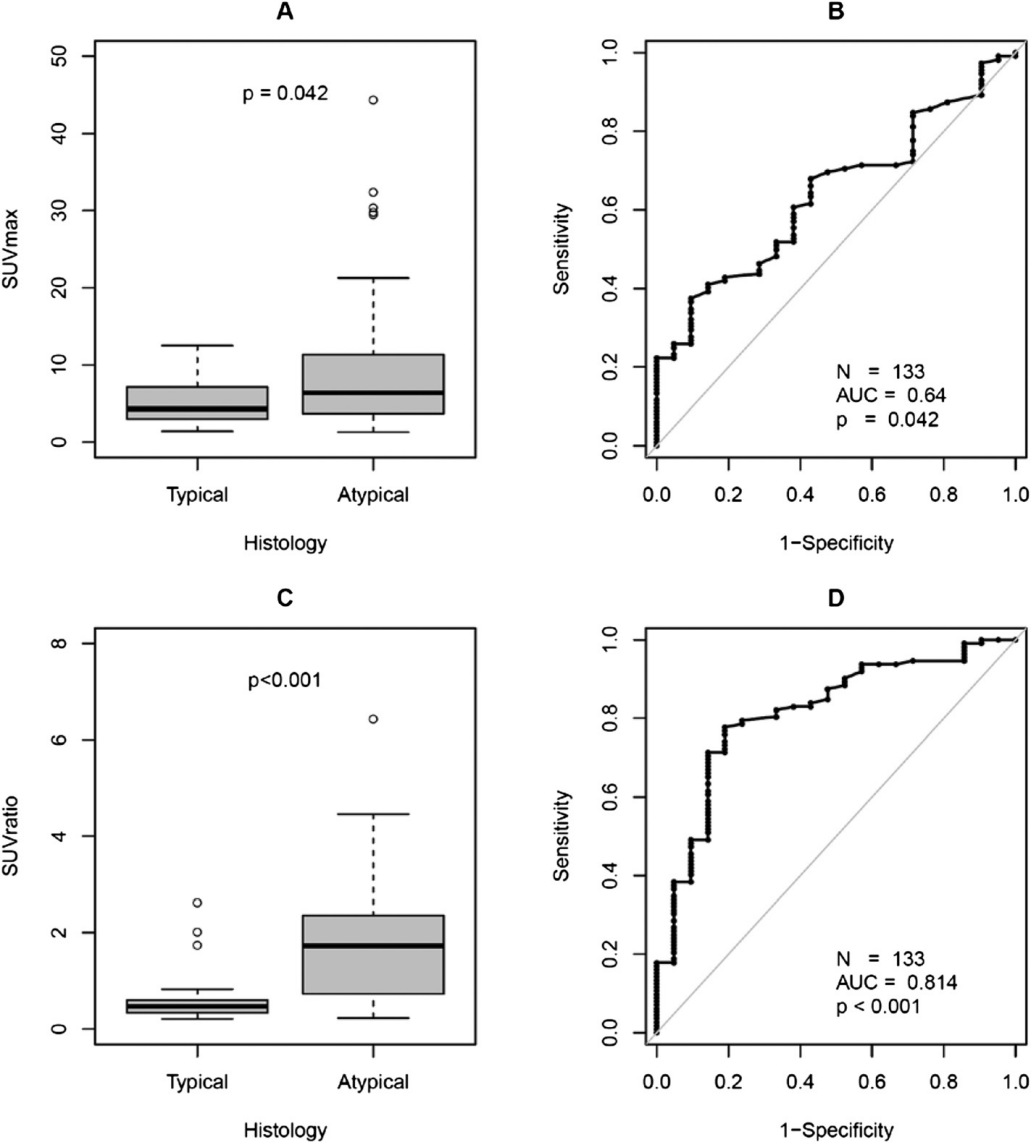
Atypical carcinoid lesions showed significantly higher somatostatin receptor expression as compared to typical carcinoid lesions of the lung (SUVmax, *p* < 0.05; SUVratio, *p* < 0.001), SUVmax (**a**) and (**b**); SUVratio (**c**) and (**d**)

SUVmax of SR-positive tumor lesions (133/186) were normalized to the SUVmax of the liver to generate normalized SUV (SUVratio) values. SUVratio was significantly higher in AC (median/IQR/range, 1.7/0.7–2.4/ 0.2–6.4) as compared to TC (median/IQR/range, 0.5/0.3–0.6/0.2–2.6; *p* < 0.001) with respect to all lesions (*N* = 133, PT 8, metastases 125; Fig. 2). AC metastatic lesions (median/IQR/range, 1.7/0.8–2.4/0.2–6.4) also showed significantly higher SUVratio as compared to TC (median/IQR/range, 0.4/0.3–0.6/0.2–2.0; *p* < 0.001).

#### PET vs. CT—patient-based analyses

##### Frequency and characteristics of metastases

The frequency of metastases in patients with AC (13/15; 86.7 %) was higher compared to patients with TC with a trend towards significance (6/12; 50 %; *p* = 0.087). In patients with AC, 4/15 had mixed lesions, 3/15 had somatostatin receptor-negative lesions, 2/15 had no detectable lesions on SR PET, whereas in the remaining 6/15, patients all the lesions were somatostatin receptor positive. In patients with TC 1/12 had mixed lesions, 1/12 had PET-negative lesions, 7/12 had no detectable lesions on SR PET, whereas in the remaining three patients, all the lesions were somatostatin receptor positive (Table 4). Frequency of patients with mixed lesions was not statistically significant between TC (1/12 = 8.3 %) and AC (4/15 = 26.7 %; *p* = 0.34). This was also true analyzing only patients with metastases (TC vs. AC, 1/6 = 16.7 % vs. 4/13 = 30.8 %; *p* = 1).

**Table 4:** Absolute and relative frequency of somatostatin receptor-positive and somatostatin receptor-negative lesions in AC and TC patients

Histopathology	All negative	All positive	Mixed lesions	No metastases	Total
TC	1 (8.3 %)	3 (25 %)	1 (8.3 %)	7 (58.3 %)	12
AC	3 (20.0 %)	6 (40.0 %)	4 (26.7 %)	2 (13.3 %)	15
Total	4 (14.8 %)	9 (33.3 %)	5 (18.5 %)	9 (39.3 %)	27

Bone metastases were present only in AC (*N* = 6) but not in TC patients, and all bone metastases were SR PET-positive lesions.

##### Effect of PET on management strategy

Additional findings on PET missed on CT lead to upstaging in four patients (AC *N* = 3; TC *N* = 1; all restaging) resulting in change in management strategy (Table 5). Two patients (1 AC, 1 TC) with liver metastases but no extrahepatic lesions were treated with transarterial embolization, and afterloading, in one patient (AC), salvage PRRT was ruled out because of stable disease in the bone, and in the fourth patient (AC), a wait-and-watch policy was applied because of low tumor burden.

**Table 5:** Patients with PET leading to correct and incorrect staging

Patient ID	Sex	Age	Histo	Ki67	Additional CT information	Additional PET information	Change in management due to PET
PET leading to correct staging							
#27^a^	M	67	AC	2	–	1 liver, 2 bone	SD bone, no salvage PRRT indicated
#8^a^	F	63	AC	3		Liver cysts	Follow-up, without intervention
#28^a^	F	34	AC	15	–	3 bone, 3 others,	Low tumor burden, wait and watch, no PRRT
#29^a^	F	68	TC	5	–	2 liver	Afterloading of liver metastases
#31^a^	F	53	AC	20	–	2 bone	TAE of liver metastases seen on CT and SR PET because of low tumor burden on bone
CT leading to correct staging							
#1^a^	F	74	AC	10	1 recurrent tumor in lung, 9 LN	–	–
#10^b^	F	58	TC	NA	8 PT	–	–
#12^a^	M	59	AC	10	5 liver	1 LN	–
#30^a^	F	50	AC	10	7 liver	–	–

*SD* stable disease, *LN* lymph nodes, *PT* primary tumor, *TAE* transarterial embolization, *PRRT* peptide receptor radionuclide therapy, *SR* somatostatin receptor, *TC* typical carcinoid, *AC* atypical carcinoid, *M* male, *F* female, *NA* not available

^a^Restaging

^b^Staging

In four patients referred for restaging (all, AC) and in one patient referred for staging (TC), additional findings on CT missed on PET lead to correct staging (Table 5). In patients referred for restaging, additional findings on PET lead to upstaging with change in management strategy in 4/22 (18.2 %) patients. In one patient (Table 5, patient #8), one of the liver lesions seen on CT was biopsied and was confirmed to be free of malignancy. All the lesions in this patient were found to be somatostatin receptor negative, and the disease was downstaged correctly by PET.

##### Patients with multiple lung nodules

Three of 27 patients (11.1 %) had multiple lung nodules and were subclassified into DIPNECH by the tumor board based on initial findings and the follow-up results. All the lung nodules diagnosed on CT were subclassified as primary tumor due to the absence of histopathological confirmation. One patient presented with nine lymph node metastases all positive on both PET and CT. However, only 6/26 (23.1 %) lung lesions range in size from 6 to 26 mm were found to be somatostatin receptor positive with very low SUVmax (Table 6) in these patients with DIPNECH.

**Table 6:** Characteristics of patients with diffuse pulmonary neuroendocrine cell hyperplasia (DIPNECH)

	Patient 3	Patient 10	Patient 25
Ki67	5 %	NA	15 %
Transformation	TC	TC	AC
Lesion size (mm)	2–18	2–26	2–26
Somatostatin receptor positive lung lesions	4/13	0/8	2/5
SUVmax	1.4–7.9	–	1.0–2.5
LN-Metastases on SR-PET and CT	–	–	9/9

Lesion size and SUVmax are described by minimum-maximum values

## Discussion

The incidence of LNET is increasing [2]. In the absence of evidence-based consensus guidelines on the management of LNET, the current standard of practice varies appreciably according to the availability of diagnostic tools: contrast-enhanced CT is standard in virtually all LNET patients often followed by somatostatin receptor scintigraphy or Ga-68 DOTATOC/DOTATATE PET/CT. There is only one study which prospectively examined the role of SR scintigraphy during the follow-up of patients after bronchial carcinoid resection [10]. Out of 16 patients enrolled, 15 had TC and 1 had AC. The authors compared CT and SR scintigraphy and found SR scintigraphy to be useful in 2/16 patients (12.5 %) whereas CT was found to be of additional benefit to SR scintigraphy in 1/16 patients; on the other hand, SR scintigraphy was found to be false positive due to co-existing sarcoidosis in one patient whereas CT was false positive for a lung nodule in another patient. Although prospective, this study comprised almost only TC patients, and there are no reliable data in AC patients available so far.

This difficulty in standardisation of imaging tools is partly attributable to the rarity as well as to the heterogeneity of LNETs. Although our study presents the results of somatostatin receptor PET/CT in the largest patient series of low- and intermediate-grade neuroendocrine tumors of the lungs so far, collected over a time period of 6 years, it comprises still a low number of patients with however well-documented histopathology including proliferation rates.

An important aspect of tumor heterogeneity of LNET is the differential somatostatin receptor expression, partially depending on tumor grade. In our study, AC patients with intermediate-grade tumors, although not significant, were found to have a higher proportion of mixed lesions, i.e., both somatostatin receptor-positive and receptor-negative lesions as compared to TC patients which had more homogeneous somatostatin receptor expression. The lack of significance could be because of the relatively low number of patients in the two subgroups as well as due to the lower frequency of metastases in TC as compared to AC. Moreover, in our patient population, proliferation rates of TC and AC metastases were significantly higher than those of the primaries which is partly due to differences between tumor clones in primary tumors and metastases [15] challenging the choice of the perfect tracer for these tumors, i.e., FDG as tracer for rather highly proliferative and high-grade tumors vs. Ga-68-labeled somatostatin receptor analogs, usually considered as tracer of choice for the well-differentiated and, thus, low- and intermediate-grade tumors. These complex inter- and intrapatient differences in the clonal behavior of the primary tumors and the metastases can theoretically be picked up only by combining different imaging tools. Indeed, in our study, only the combination of both functional SR PET imaging and morphological contrast-enhanced CT imaging yielded the maximum information necessary for appropriate staging and restaging because concordant results between SR PET and CT were observed in only 54 % of the lesions. This rather low concordance between both imaging modalities clearly shows the need for combining both with each other to SR PET/contrast-enhanced (ce) CT.

In general, CT was more sensitive for staging of liver and lung lesions whereas PET performed significantly better in the detection of bone metastases. Lower sensitivity of PET in the detection of lung lesions as well as liver lesions as compared to CT is at least partly be attributable to the partial volume effect below 1 cm in diameter, normal physiological uptake of Ga-68 DOTATOC/DOTATATE in liver as well as to breathing movement artefacts [16]. In one patient, the disease in the liver was classified to be polycystic liver disease. In this patient, the hypodense lesions in the liver were all somatostatin receptor negative thereby making it essential to keep this as differential diagnosis in patients with neuroendocrine tumor and somatostatin receptor-negative lesions.

In contrast, additional lesions were detected by PET in 3 AC patients (patient 27, 1 liver lesion, 2 bone lesions; patient 28, 3 lymph node metastases, 3 bone lesions; patient 31, 2 bone lesions) and 1 TC patient (patient 29, 2 liver lesions). More importantly, in patients referred for restaging, additional findings on PET lead to upstaging with change in management strategy approximately every fifth patient.

**Fig. 3:**
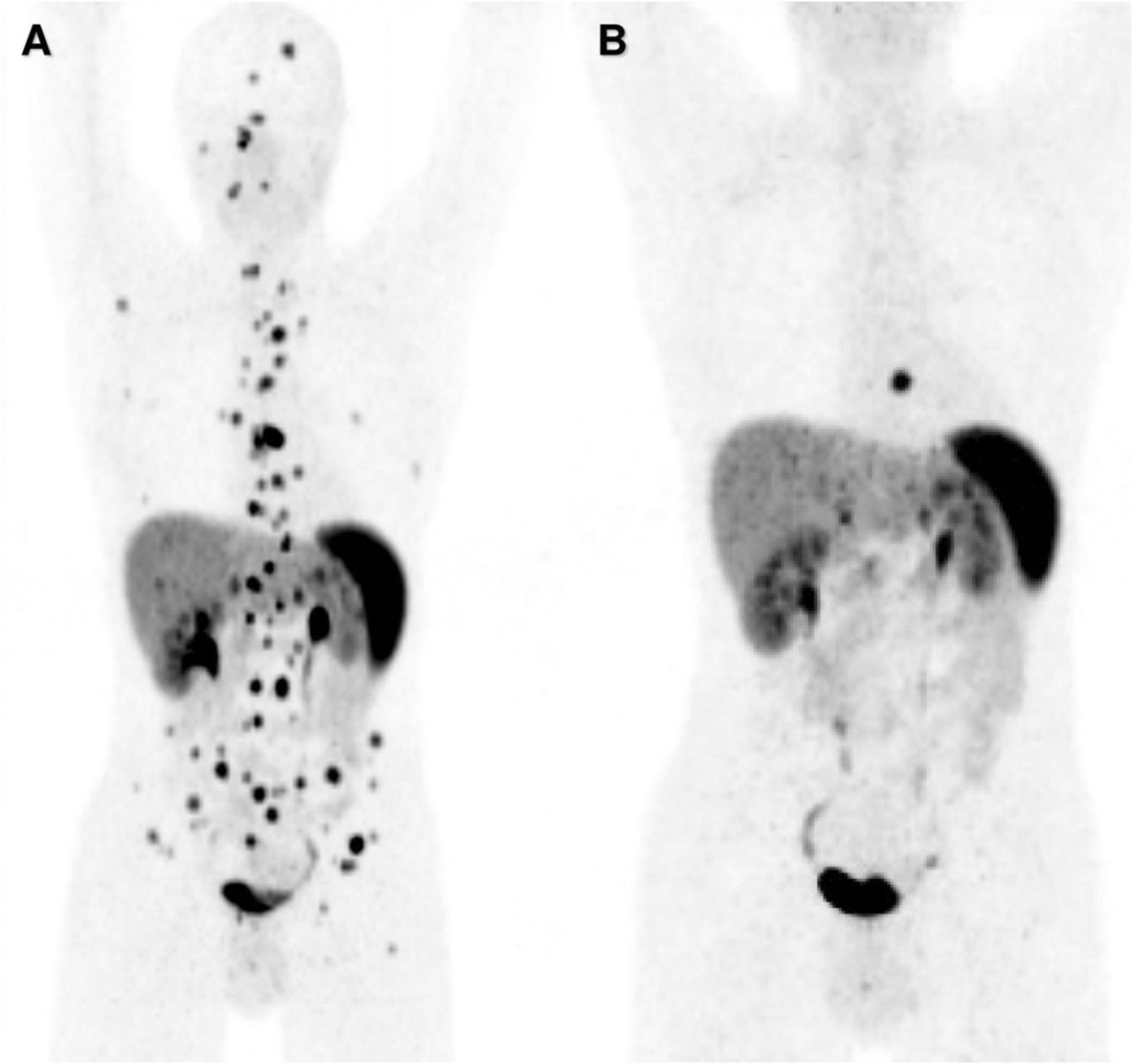
Atypical carcinoid patient referred for restaging with Ga-68 DOTATOC PET/CT and was treated with 3 cycles of peptide receptor radionuclide therapy (PRRT; 2 cycles of Y-90 DOTATOC and 1 cycle of Lu-177 DOTATATE) showing excellent response. **a** Ga-68 DOTATOC Maximum intensity projection images before PRRT. **b** Ga-68 DOTATOC maximum intensity projection images 22 months after first PRRT

Apart from allowing correct staging and restaging, combined SR-PET/CT allows selection of appropriate patients for PRRT by ruling out mixed lesions which is a contraindication for performing PRRT and by allowing quantification of somatostatin receptor expression and assessment of SR-positive tumor burden which is required for making a decision on PRRT. In the absence of standardized systemic treatment option for AC and TC patients, PRRT appears to be a valuable therapeutic option. In our overall patient population (data not shown here), three AC patients SR-PET/CT showed very high somatostatin receptor expression and no mismatch between PET and CT results. These patients were treated with PRRT, and an excellent therapy response could be shown in one patient (see Fig. 3).

The treatment strategy of LNETs also depends on their metastasizing potential. Our observation that TC metastasizes less frequently as compared to AC is in line with previous studies: this difference is related to their differences in proliferative activity and, thus, aggressiveness, with AC having a higher frequency of nodal (50 %) and distant metastases (20 %) as compared to TC [17, 18]. However, typical carcinoids can also metastasize as shown in our retrospective analyses in which PET/CT revealed metastases in 50 % of the patients, making it mandatory to perform SR PET/CT in patients with TC at least once for staging/restaging to rule out distant metastases. In the presence of somatostatin receptor-negative lesions during initial staging with SR PET/CT, further follow-up examinations should then be based on clinical symptoms, CT, and serum tumor markers while SR PET/CT should be considered for follow-up examinations in patients with receptor-positive lesions.

For atypical carcinoids, especially in cases with high suspicion of tumor recurrence after surgery and/or higher grade tumors, alternatively FDG PET/CT could be performed in case of SR PET-negative lesions as is supported by the study of Kayani et al. [19] who found higher grade LNET to be more FDG avid as compared to Ga-68 DOTATATE. Of special note, they found FDG PET to be less useful in the differentiation of post-radiation changes from vital tumor tissue.

**Fig. 4:**
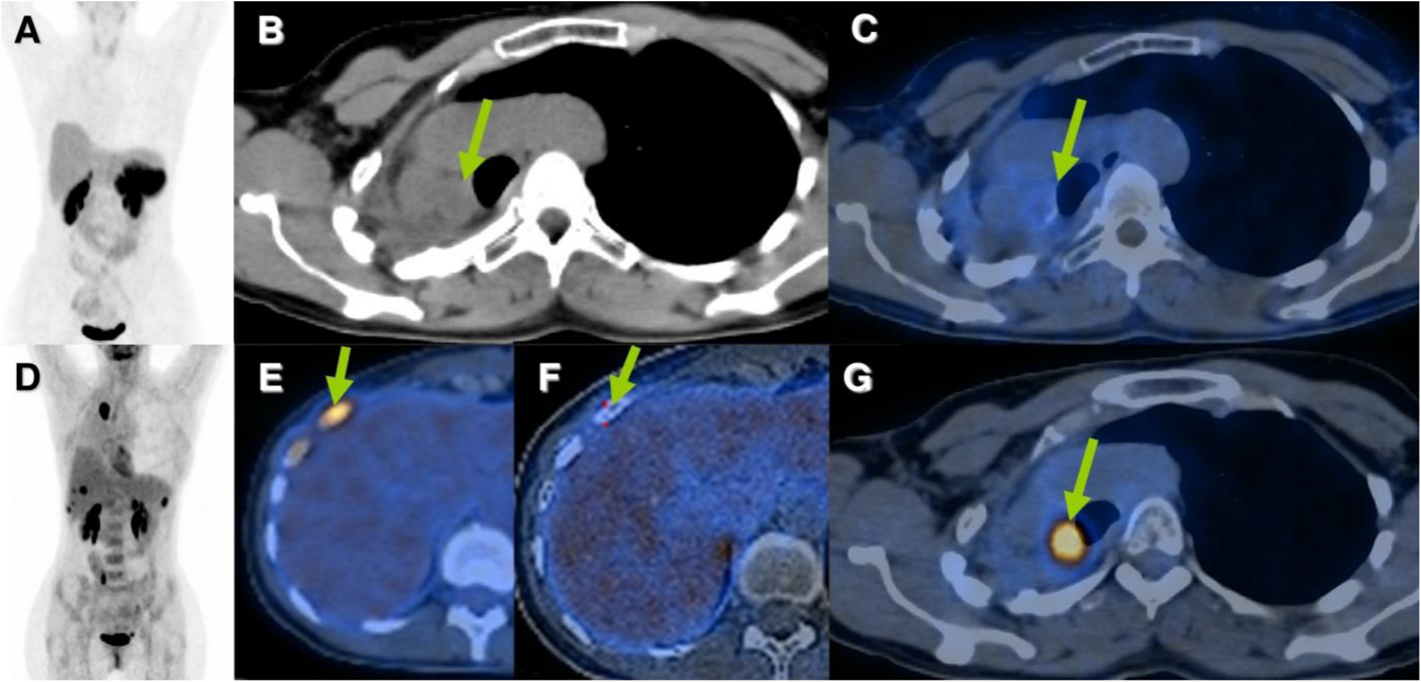
Atypical carcinoid of the lung, first diagnosed in 03/2000. Following upper and middle lobe resection of the right lung (03/2000) and external beam irradiation therapy with 70 Gy (06/2007), the patient underwent multiple operations for residual tumor. Patient was referred for restaging with Ga-68 DOTATOC and FDG PET/CTs (result not discussed in the text). Both the PET/CTs performed in 2010 showed somatostatin receptor-negative, FDG-positive local residual tumor and bone metastases. **a** Ga-68 DOTATOC PET MIP image. **b** CT axial slides. **c**, **f** Ga-68 DOTATOC PET/CT fused axial images. **g**, **e** FDG PET/CT fused axial images. **d** FDG PET MIP image

Surgery is generally offered with curative intent to all patients with operable well-differentiated metastases from NET regardless of the site of origin (foregut, midgut, or hindgut) [20]. The majority of patients will have recurrent disease at 5 years if distant metastases were present at initial diagnosis [20]. One of the patients in our retrospective analysis presented with local recurrence 10 years after the first surgical resection (Fig. 4). Occurrence of late metastases in patients with carcinoid lung tumors has been already previously reported and necessitates regular follow-up of such patients for at least 10 years [21] and probably even longer.

Tumor recurrence can either be hepatic and/or extrahepatic. Liver is the most frequent site of distant metastases. Prior to liver surgery with curative intent, it is important to rule out extrahepatic metastases. High rates of detection of bone and lymph node metastases by SR PET suggest that at least one combined SR PET/CT should be performed in patients with AC and TC prior to any liver surgery. However, if liver surgery is planned, MRI with liver-specific contrast agent should always be performed in addition to CT and/or SR PET/CT because it has the highest sensitivity for detection of liver metastases [22]. One of the inherent limitations of SR PET in detection of liver metastases is the normal physiological uptake of the tracer in hepatocytes which lead to relatively low target non-target ratio, especially if the lesions are smaller than 1 cm or if the lesions have low somatostatin receptor expression.

**Fig. 5:**
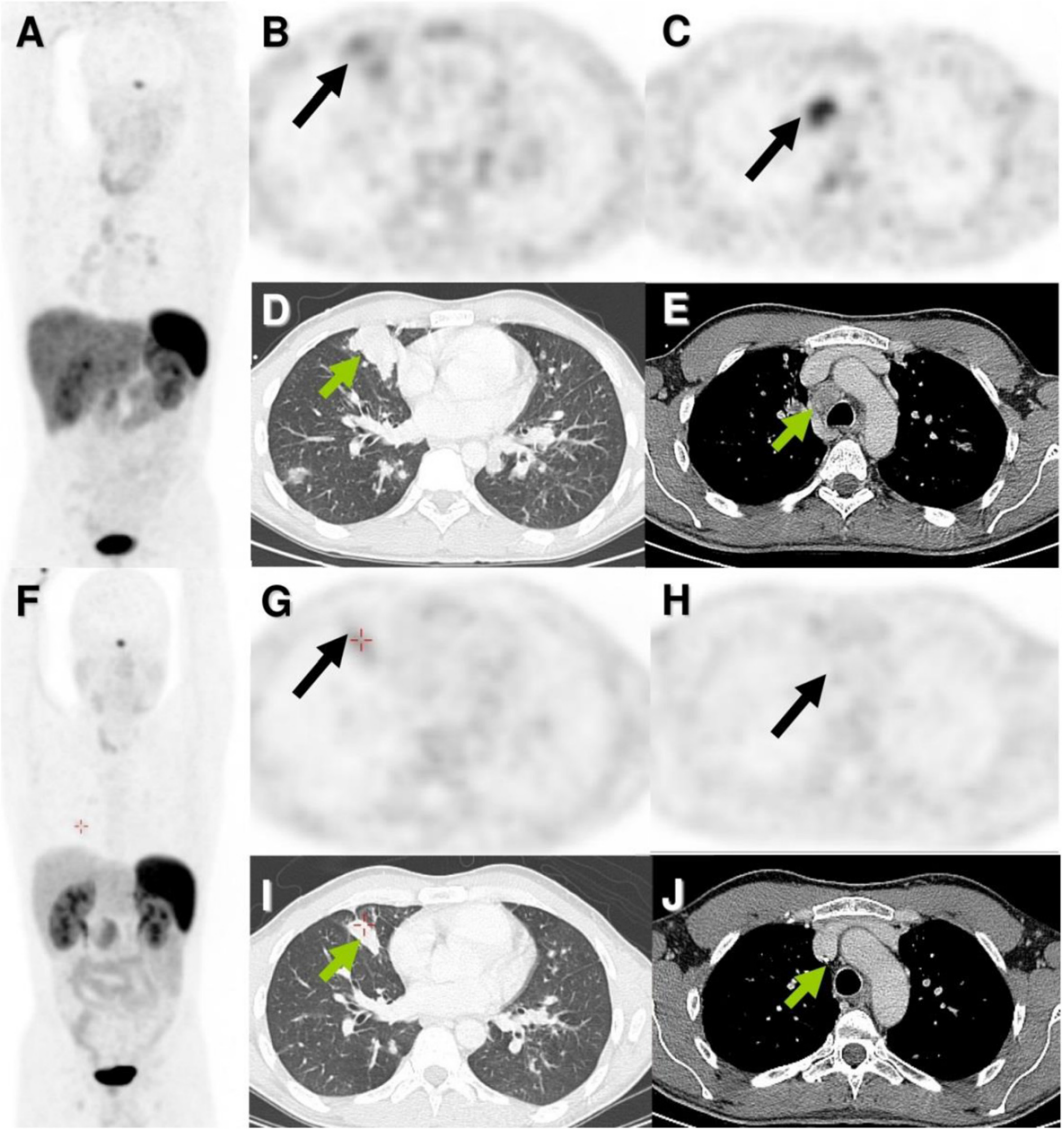
Patient with initially diffuse pulmonary neuroendocrine cell hyperplasia (DIPNECH) with transformation into an atypical carcinoid was referred for Ga-68 DOTATOC PET/CT. Based on weak somatostatin receptor expression, patients was treated with chemotherapy (folinic acid, 5 fluorouracil, and oxaliplatin) and showed a good response. **a**-**e** Before chemotherapy. **f**-**j** After chemotherapy. **a**, **f** Maximum intensity projection PET images. **b**, **c**, **g**, **h** Axial PET images. **d**, **e**, **i**, **j** Corresponding axial CT images. Partial remission of the mildly receptor positive lesion in the right lung is well appreciated on CT (*arrows*). On MIP images, the previously receptor-positive hilar and mediastinal lymph node lesions also show response to treatment

On the other side of the spectrum of lung neuroendocrine neoplasms, as far as receptor expression and mismatch between SR PET and CT results is concerned, are the DIPNECH. Management of patients with DIPNECH has always posed a major challenge because very little is known about their exact biological behavior and clinical course [5, 7]. In our analysis, we identified three patients with malignant transformation of initial DIPNECH into TC or AC. One of these patients also developed lymph node metastases and later on responded to chemotherapy underscoring the need of routine follow-up in this rare type of lung tumors (Fig. 5).

Our study is not the first one to look into the role of Ga-68-labeled somatostatin receptor PET/CT in LNET. Previous studies have compared Ga-68-DOTATATE and Ga-68-DOTATOC in comparison to FDG PET/CT in patients with AC and TC [19, 23, 24]. In these studies, the main purpose was to look at the different rates of somatostatin receptor expression in TC and AC. We also looked at the degree of somatostatin receptor expression in TC and AC. Our findings, however, are contradictory to the previously reported results [19, 23, 24]. In our analysis, TC lesions were found to have a significantly lower tumor SUVmax and SUVratio than AC lesions (Fig. 2) whereas the previously published studies reported significantly higher SUVmax in TC as compared to AC [19, 23, 25]. This difference could be primarily due to the difference in the patient populations. While in our analysis, most of the patients (22/27; 81.5 %) underwent SR PET/CT for restaging after primary tumor resection, in the study from Kayani et al. [19], 83 % (15/18) of the patients underwent SR PET/CT for staging, and the study of Venkitaraman et al. [23] considered only patients (*N* = 26) referred for staging. Furthermore, the ratio of TC (44 %) vs. AC (56 %) in our population is quite different in comparison to Kayani’s group [19] with 72 % TC (*N* = 11) vs. 11 % AC (*N* = 2) or Venkitaraman et al. [23] (TC = 81 %, *N* = 21 vs. AC 19 %, *N* = 5). In their analyses of SUV in TC and higher grade LNET, Kayani et al. [19] categorized SCLC and NSCLC with NET differentiation in one group and LCNEC together with AC into another group of NEN which is not in accordance with the WHO classification [4] and is also distinct from the classification suggested by Rindi et al. [14]. Rindi et al. [14] included information on findings by SR scintigraphy in three patients with TC and five patients with AC and found a higher incidence of negative scintigrams in TC as compared to AC (33 % vs. 20 %). Thus, our findings, based on a larger patient population, confirm these initial results by Rindi et al. showing that somatostatin receptor expression is also a valuable biomarker for tumor detection and (re) staging in patients with intermediate grade AC tumors.

One of the major limitations of this analysis is its retrospective nature. Although we included all patients with AC and TC which received SR PET/ceCT at our ENETS center in this analysis, there will probably be a selection bias due to the fact that rather patients with suspicion for relapse or metastatic disease will have been referred for SR PET/CT. Thus, our patient population may not represent the full, i.e., unselected cohort of patients with AC and TC, and thus, the distribution of imaging characteristics of our patients might be biased to some extent. Apart from this, we used in our study both radiotracers, Ga-68 DOTATOC and Ga-68 DOTATATE, for SR PET imaging. The use of either tracer was solely based on its availability due to patent constraints but not on medical reasons. Although these tracers have slightly different binding affinities to somatostatin receptor subtypes, there seems to be no clinically relevant difference in the diagnostic accuracy for NET [20]. However, it would be interesting to also look into other somatostatin receptor analogs covering a broader spectrum of somatostatin receptor subtypes such as Ga-68 DOTANOC [26].

## Conclusions

In conclusion, TC and AC patients have complex patterns of metastases which make it necessary to combine functional, i.e., Ga-68 SR PET and morphological imaging, i.e., contrast-enhanced CT for appropriate restaging because only 54 % of the lesions are concordantly detectable by both modalities. The major advantage of SR PET lies in the detection of additional bone lesions. Of similar importance, SR PET/CT allows correct discrimination of patients with heterogeneous (mixed lesions) and homogeneous (all lesions are either somatostatin receptor-positive or somatostatin receptor-negative) lesions which is an essential prerequisite for the selection of the appropriate therapy, especially with respect to PRRT. In patients referred for restaging SR, PET may have a relevant impact on treatment strategy in up to 18 % of patients with typical and atypical lung carcinoids.
